# Comparative Metabolomic Analysis of the Cambium Tissue of Non-transgenic and Multi-Gene Transgenic Poplar (*Populus* × *euramericana* ‘Guariento’)

**DOI:** 10.3389/fpls.2018.01201

**Published:** 2018-08-17

**Authors:** Kun Ning, Changjun Ding, Wenxu Zhu, Weixi Zhang, Yufeng Dong, Yingbai Shen, Xiaohua Su

**Affiliations:** ^1^State Key Laboratory of Tree Genetics and Breeding, Research Institute of Forestry, Chinese Academy of Forestry, Beijing, China; ^2^Key Laboratory of Tree Breeding and Cultivation, State Forestry Administration, Beijing, China; ^3^College of Forestry, Shenyang Agricultural University, Shenyang, China; ^4^Shandong Provincial Key Laboratory of Forest Tree Genetic Improvement, Shandong Academy of Forestry, Jinan, China; ^5^College of Biological Sciences and Technology, Beijing Forestry University, Beijing, China; ^6^Co-Innovation Center for Sustainable Forestry in Southern China, Nanjing Forestry University, Nanjing, China

**Keywords:** metabolomics, multi-gene transgenic poplar, cambium, GC–MS, UPLC–MS/MS

## Abstract

Poplar, a model for woody plant research, is the most widely distributed tree species in the world. Metabolites are the basis of phenotypes, allowing an intuitive and effective understanding of biological processes and their mechanisms. However, metabolites in non-transgenic and multi-gene transgenic poplar remains poorly characterized, especially in regards of the influences on quantity and in the analysis of the relative abundance of metabolites after the introduction of multi stress-related genes. In this study, we investigated the cambium metabolomes of one non-transgenic (D5-0) and two multi-gene (*vgb, SacB, ERF36, BtCry3A*, and *OC-I*) transgenic lines (D5-20 and D5-21) of hybrid poplar (*Populus* × *euramericana* ‘Guariento’) using both gas chromatography–mass spectrometry (GC–MS) and ultra-performance liquid chromatography-tandem mass spectrometry (UPLC–MS/MS). We aimed to explore the effects of the exogenous genes on metabolite composition and to screen out metabolites with important biological functions. Finally, we identified 239 named metabolites and determined their relative abundance. Among these, 197 metabolites had a different abundance across the three lines. These methabolites spanned nine primary and 44 secondary metabolism pathways. Arginine and glutamate, as substrates and intermediates in nitrogen metabolism, and important in growth and stress-related processes, as well as sucrose, uridine diphosphate glucose, and their derivatives, precursors in cell wall pathways, and catechol, relevant to insect resistance, differed greatly between the genetically modified and non-transgenic poplar. These findings may provide a basis for further study of cambium metabolism, and fully understand metabolites associated with stress response.

## Introduction

In gymnosperms and dicotyledons, the cambium, a thin layer of ubiquitous active lateral meristem, promotes the continuous thickening of plants providing partially undifferentiated cells for plant growth. Moreover, it forms parallel rows of cells, which result in secondary tissue (Guo et al., 2009). Cambium is vital to the growth and development of woody plants. Like most other pioneer species, poplar is characterized by rapid growth and can quickly colonize open areas (Broeck, 2003). However, drought, high salinity, flooding, pests, diseases as well as biotic and abiotic stresses affect the yield and quality of poplar (Bartels and Sunkar, 2005; Ferry et al., 2006; Sairam et al., 2008). Previous works created numerous transgenic trees with the ability to resist adverse environmental stresses. Nevertheless, simultaneous enhancement of multiple characteristics continues to be a matter of great urgency and practical significance in woody species.

In a previous study, we generated a multi-gene transgenic poplar (*Populus* × *euramericana* ‘Guariento’), harboring five exogenous stress-related genes [*vgb*, encoding *Vitreoscilla* hemoglobin (VHb); *SacB*, encoding levansucrase related to fructan biosynthesis in *Bacillus subtilis*; *JERF36*, encoding jasmonate/ethylene-responsive factor protein from tomato; *BtCry3A*, encoding δ-endotoxin from *Bacillus thuringiensis* and *OC-I*, encoding the proteinase inhibitor oryzacystatin I from rice], co-transferring them into poplar (*Populus* × *euramericana* ‘Guariento’) using biolistic bombardment (Wang et al., 2007). Transcriptome analysis also found more differential expressed genes related to stress responses in the transgenic line D5-20 (Zhang et al., 2014). However, how these transferred multigene activate and change the plant metabolome to cope with stressors is still unknown.

Compared with the substantial achievements of whole genome re-sequencing and RNA sequencing, metabolomics continues to lag behind in tree biology (Robinson et al., 2017). Metabolomics is a valuable tool for comprehensive, non-biased, and high-throughput analysis of complex metabolite in specific organisms or environmental stressors, and enhances the understanding of the mechanisms behind biological responses (Gibbons et al., 2015; Meijón et al., 2016). Nowadays, the combination of MS-based platforms and other analytical technologies can increase metabolome coverage (Escandón et al., 2018). Gas chromatography–mass spectrometry (GC–MS) can measure the majority of primary metabolites, whereas liquid chromatography–mass spectrometry (LC–MS) can better cover large hydrophobic metabolites predominant in secondary metabolisms (Doerfler et al., 2013). For example, this allowed for the comparison of the metabolome of genetically modified and non-genetically modified soybean seeds for biosafety assessment (Clarke et al., 2013). Moreover, as a sugar-metabolizing enzyme, UDP-glucose pyrophosphorylase (UGPase) catalyzes the reversible reaction of UDP-glucose and pyrophosphate, metabolic profiling of xylem, phloem, and leaves of *PdUGPase2-*overexpressing *Populus deltoides* using GC–MS showed that *PdUGPase2* disrupts the primary and secondary metabolisms, reduces the levels of sugar and starch, and increased phenolic compounds (Payyavula et al., 2014).

At present, the study of plant metabolites explores plant systemic response to genetic and environmental changes to react to adverse environmental stress, and to improve crop yield and quality (Saito and Matsuda, 2010; Adamski and Suhre, 2013). Furthermore, it is an important research tool for screening crop cultivars and evaluating transgenic plants (Keurentjes, 2009; Yang et al., 2014). Many metabolic studies focus on agricultural crops, yet large scale metabolomic studies in forestry are lacking. Furthermore, non-targeted metabolic profile analysis between non-transgenic and transgenic perennial poplar using ultra-performance liquid chromatography-tandem mass spectrometry (UHLC/MS/MS) or GC/MS have been rarely reported in woody plants, especially to investigate metabolic adjustments in plant after the transformation of multiple genes. Here, we conducted a large-scale non-targeted metabolomic analysis of the cambium of two transgenic and one non-transgenic lines of poplar. Our findings may identify metabolic markers for plant stress response.

## Materials and Methods

### Plant Material and Cambium Collection

One non-transgenic (D5-0) and two transgenic 7-year-old poplar lines (D5-20 and D5-21), respectively, were grown under natural conditions at the Shou Guang test forest of the Chinese Academy of Forestry in Shandong, China. During the most rapid seasonal growth stage, 18 cambium tissue samples (six biological replicates per line) were scraped with a knife and removed from the tree bark 3 m from the ground. The samples were quickly frozen in liquid nitrogen and stored at –80°C.

### Metabolite Profiling

Metabolic profiling of poplar cambium was performed in SJTU-Metabolon Joint Metabolomics Laboratory using a global unbiased platform. More detailed information about the instrument, data acquisition and processing, and metabolite identification and quantitation, were published elsewhere (Evans et al., 2009; Ohta et al., 2009). For sample extraction, cambiums of each sample (six biological replicates each sample) were ground into a fine powder in liquid nitrogen using a SPEX 6870 Freezer/Mill (SPEX SamplePrep, Metuchen, NJ, United States), and then lyophilized in a vacuum-freeze dryer (FreeZone Freeze Dry System, Labconco, KS, United States). Lyophilized powder (40 mg) of each sample was extracted at room temperature using 400 μl methanol extraction buffer containing recovery standards (Qu et al., 2014).

For GC/MS analysis, a gas chromatography system (CP-3800 Varian, Inc.) that equipped with a CP-8400 automatic injector and a mass spectrometry detector system of 4,000 electron impact ion trap were employed herein. A VF-1ms capillary column (30 m × 0.25 mm × 0.25 μm) was used with a constant flow rate at 1.0 mL/min of helium as carrier gas. Samples were re-dried under vacuum desiccation for a minimum of 24 h, and then derivatized using BSTFA (bistrimethyl-silyl-triflouroacetamide) in dried nitrogen. The derivatized samples were transferred to autosampler vials (2 mL GC), and each sample of 1 μL was injected into GC–MS with the 25:1 split ratio. The GC column was 5% phenyl and the initial oven temperature was kept at 100°C for 2 min, then with a rate of 10°C/min to increase 300°C and remained at 300°C for 10 min. The temperatures of the injector and transfer pipeline were set at 250°C (Jung et al., 2013). The metabolites were separated by chromatographic column with a time gradient elution and then detected by mass spectrometer, the retention time was the peaking time of chromatography. Electron impact ionization with 70 eV ionization energy and full scan in the range of 50–1,000 m/z were used to analyze the samples on a Thermo-Finnigan Trace DSQ fast-scanning single-quadrupole mass spectrometer. The retention time and molecular weight (m/z) for all detectable ions were measured.

The UPLC/MS/MS platform was based on a Waters Acquity UPLC (Waters, Milford, MA, United States) and a linear trap quadrupole (LTQ) mass spectrometer (Thermo Fisher, Corporation), which had a linear ion-trap (LIT) front end and a Fourier transform ion cyclotron resonance (FT-ICR) mass spectrometer backend. The extract samples were split into two aliquots, dried and then reconstituted in acidic or basic LC-compatible solvents, and each solvent contained 11 or more internal standards at fixed concentrations (Chen et al., 2016). The acidic extract samples of one aliquot was analyzed for positive ions, while the other basic extract samples was analyzed for negative ions in two independent injections using separate acid/base dedicated 2.1 mm × 100 mm Waters BEH C18 1.7 μm particle columns heated to 40°C. Extracts reconstituted in acidic conditions were gradient eluted at 350 μl/min using (a) 0.1% formic acid in water and (b) methanol containing 0.1% formic acid (0 to 70% b in 4 min, 70–98% b in 0.5 min, and 98% b for 0.9 min), while the extracts reconstituted in ammonium bicarbonate used: (a) 6.5 mM ammonium bicarbonate in water, pH8.0 and (b) 6.5 mM ammonium bicarbonate in 95/5 methanol/water (the same gradient profile as above) at 350 μl/min (Evans et al., 2009). The mass spectrometry analysis was performed alternately between full MS and data-dependent MS/MS scans using dynamic exclusion.

The metabolites were identified by automatically matching ion features against the Metabolon’s reference library entries. The reference library of each platform was generated from approximately 1,500 authentic standards and each library contains retention time index (RI), molecular ion mass (m/z), and MS/MS spectra data on all molecules as well as their associated adducts, in-source fragments, and multimers up to ∼10,000 recorded (Evans et al., 2009). The combination of chromatographic retention index and mass spectrum characteristics showed an indication to match the specific metabolite.

### Data Extraction and Statistical Analysis

Data normalization was performed for internal consistency by processing a constant weight per volume of extraction solvent for each sample, and each compound was corrected by registering the medians to equal one (1.00) and normalizing each data point proportionately in run-day blocks. For UPLC/MS/MS platform, accurate mass determination could be performed for the ions with counts greater than two million and made on the parent ion as well as fragments for that the typical mass error less than 5 ppm. Peaks were identified using Metabolon’s proprietary peak integration in-house software, and component parts were stored in a separate and specifically designed complex data structure (Chen et al., 2016). The one-way analysis of variance (ANOVA) was used to determine the significant differences between the means of tested groups. Classification analysis was conducted using random forest analyses. Principle component analysis was carried out using SIMCA-P 12.0 software. *p*-Values and False discovery rate (FDR) of each correlation were calculated using Cor.test function (Benjamini and Yekutieli, 2001). A *p*-value of <0.05 was considered statistically significant. FDR was conducted to correct multiple comparisons, while the FDR for a given set of compounds can be estimated using the *q*-value (Storey and Tibshirani, 2003).

## Results

### Metabolomic Profiling of Poplar Cambium

**FIGURE 1:**
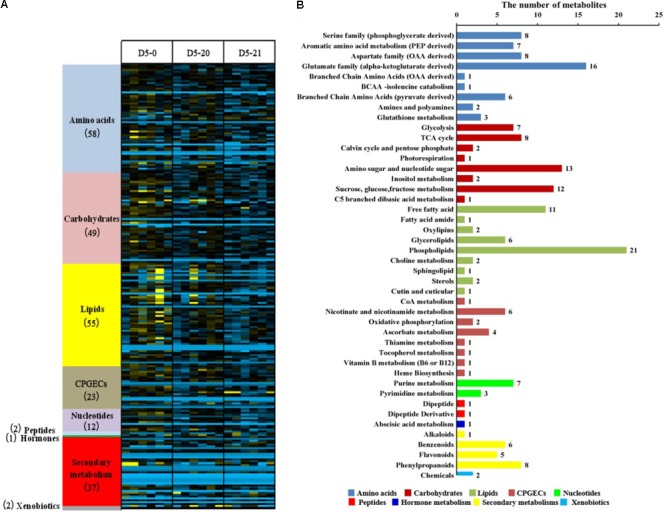
Distribution of identified metabolites in the cambium of non-transgenic and transgenic poplar. **(A)** Heat map showing the clustering of detected metabolites into nine classes across the three lines. Different colors represent nine metabolic pathways of the 239 metabolites, and the numerals in parentheses represent the numbers of metabolites identified in the primary metabolic pathways. **(B)** The distribution of metabolic pathways of the differential 197 metabolites. Ordinate reports the various metabolic pathways, while abscissa represents the number of metabolites. Different colors represent primary pathways.

We investigated the metabolome of poplar cambium using an untargeted global metabolomic platform that integrated GC/MS and UPLC/MS/MS to evaluate the differences between non-transgenic and transgenic lines. We identify 239 metabolites confirmed by reference standards, and we also determined the relative abundance of metabolites. We constructed a heat map to display the scaled data, arranging compounds by pathway groups. According to annotations in PlantCyc^1^ and in the Kyoto Encyclopedia of Genes and Genomes (KEGG Compound Database^2^), the identified metabolites mapped to general biochemical pathways (Dersch et al., 2016). These metabolites divided into nine primary pathways (**Figure 1A**), which contained to 48 sub-pathways (**Supplementary Table 1**). Forty-two metabolites showed no significant difference across the three lines. These pathways mapped to most of the primary and secondary metabolisms. We found differences between the six biological replicates, especially in line D5-0. Variation between biological replicates within lines tended to be quite high, often as high as the average variation between lines.

### Metabolic Variations Between Non-transgenic and Transgenic Poplar

We excluded metabolites which showed no differences between the three lines, obtaining 197 out of 239 metabolites. These were part of nine primary metabolisms and 44 secondary metabolism pathways, including 52 amino acids, 47 lipids, 46 carbohydrates, 20 secondary metabolisms, 17 CPGECs (cofactors, prosthetic groups, electron carriers), 10 nucleotides, two xenobiotics, two peptides, and one hormone metabolism (**Figure 1B** and **Supplementary Table 2**). Notably, primary metabolisms involving amino acid, lipids, and carbohydrates account for the 73.6% of the total metabolites and mainly participated in the pathways.

**FIGURE 2:**
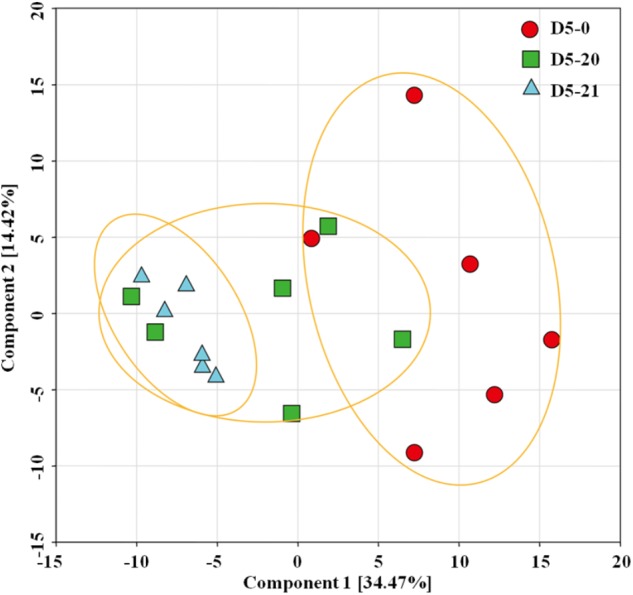
Principal component analysis (PCA) score plots of metabolome data from the cambium of transgenic and non-transgenic poplar. PCA score plot of the three lines. The red circle indicates non-transgenic line D5-0, while the green square and blue triangle indicate multi-gene transgenic line D5-20 and D5-21, respectively. The plot displays PC1 on the ordinate axis and PC2 on the abscissa.

To reduce the complexity of the metabolomics data, we conducted an unsupervised multivariate data analysis method principal component analysis (PCA) of the metabolites showing differences between the different lines (**Figure 2**). Our PCA analysis with two principal components explaining 48.89% of the overall variance of the metabolite profiles, 34.47 and 14.42% for principal component 1 (PC1) and PC2. The PC scores revealed that compounds from D5-0 clustered separately from both those from D5-20 to D5-21, with only a partial overlap to the compound of line D5-20. Line D5-20 and D5-21 instead clustered together. Line D5-21 was most consistent, and was clearly separated from D5-0.

To further identify metabolic variation across the cambium of the three lines, we performed a one-way ANOVA (**Table 1**), where we considered a *p*-value ≤ 0.05 significant. We observed significant differences between non-transgenic and transgenic poplar. In the comparison between D5-20 and D5-0, we identified a total of 52 significant metabolic changes, containing 20 carbohydrates, nine lipids, seven amino acids and derivatives, seven CPGECs, five secondary metabolites, and four nucleotides, of which five metabolites were increased and 47 metabolites were decreased in line D5-20. In the comparison between D5-21 and D5-0, we observed 83 significant metabolic changes, including 25 amino acids and derivatives, 21 carbohydrates, 17 lipids, eight nucleotides, six CPGECs, four secondary metabolites, and two peptides. Nine of the metabolites were increased and 74 metabolites were decreased in line D5-21. Finally, for the comparison between D5-21 and D5-20, we identified 13 significant metabolic changes, covering one amino acid, three carbohydrates, four lipids, three nucleotides, one peptide, and one secondary metabolite. Two of the metabolites were increased, while 11 metabolites were decreased in line D5-21.

**Table 1:** Analysis of the differences metabolite content between transgenic and non-transgenic poplar by ANOVA.

Ratio	Sig meet criteria	Sig up	Sig down
D5-20/D5-0	52	5	47
D5-21/D5-0	83	9	74
D5-21/D5-20	13	2	11

### Metabolic Changes in Transgenic Poplar

When we compared D5-20 and D5-21 to D5-0, metabolites that showed changes were primarily involved in metabolisms of amino acids, carbohydrates, lipids, and secondary metabolites. In the amino acid primary pathway, arginine and glutamate were significantly increased in D5-20 and D5-21. However betaine, 2-hydroxyadipate, 2-pyrrolidinone, and leucine were reduced. The metabolites of carbohydrate metabolic pathways were closely related to glycolysis, citric acid cycle (TCA cycle), and the metabolisms of amino sugar, nucleotide sugar, sucrose, glucose, and fructose. The two carbohydrates which showed the greatest increase were uridine diphosphate glucose (UDP-glucose), and sucrose in D5-20 and D5-21, while the five carbohydrates which showed the greatest decrease were glucuronate, malate, *N*-acetylglucosamine, ribose, and xylose. In the lipid primary pathway, beta-sitosterol, sphinganine, 2-hydroxyglutarate, 1-stearoylglycerol (1-monostearin), 1-stearoylglycerophosphocholine (18:0), 1-oleoylglycerol (1-monoolein), and 12,13-DiHOME were reduced. In the secondary metabolic pathway, catechol was the most significantly increased in both transgenic poplar, while 4-hydroxybenzoate and 4-hydroxycinnamate were markedly reduced in the transgenic poplar lines. In addition, five metabolites grouped under CPGECs and four metabolites from the nucleotide primary pathways decreased in transgenic lines, the greatest changes occurred in nicotinate/nicotinamide metabolism and purine metabolism. Altogether, compare to non-transgenic line D5-0, we have screened out five significantly increased metabolites in two multi-gene transgenic lines, including two alpha-ketoglutarate derived in amino acid super pathway (arginine and glutamate), two carbohydrates (UDP-glucose and sucrose), and one secondary metabolisms (catechol) (data listed in **Supplementary Table 2**).

### Specific Pathway Related Functional Effects

**FIGURE 3:**
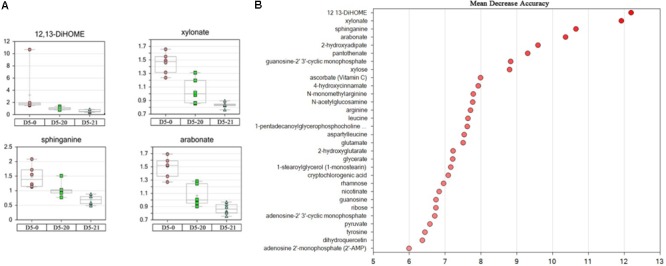
Four most important compounds and top 30 metabolites. **(A)** The four most important compounds for correct classification of MDA value > 10 from non-transgenic and transgenic polar. The ordinates represent scaled intensity. **(B)** The top 30 metabolites for classification.

We used random forest analysis (RF) to assess which compounds contributed most to differentiation between lines. Lipids and pentose derivatives tended to dominate the separation of the cambium samples. The oxylipin 12,13-DiHOME and the pentose sugar acid xylonate, sphinganine, a lipid possibly involved in cutin synthesis, and the pentose sugar acid arabonate were the four most important compounds for correct classification of cambium from each line (**Figure 3A**).

The probability that the samples were correctly classified was 72% (**Table 2**). When calculating the “important variable,” we used “Mean Decrease Accuracy-MDA” to identify the variables (metabolites) that contributed the greatest to the classification. We sorted RF values by the “importance” of metabolites. **Figure 3B** and **Table 3** show the first 30 metabolites and their correct classification.

**Table 2:** Random forest analysis used for the analysis of the differences in metabolite content between transgenic and non-transgenic poplar.

Group		Predicted			Error rate
		D5-0	D5-20	D5-21	
Actual	D5-0	5	1	0	0.17
	D5-20	1	3	2	0.50
	D5-21	0	1	5	0.17
Out of bag error rate		—	—	—	0.28

**Table 3:** Top 30 metabolites that contributed significantly to the correct classification of D5-20 and D5-21 compared to D5-0.

Biochemical name	Fold of change		*p*-Value		*q*-Value		Super pathway
	D5-20/D5-0	D5-21/D5-0	D5-20/D5-0	D5-21/D5-0	D5-20/D5-0	D5-21/D5-0	
12,13-DiHOME	0.32	0.19	0.0392	0.0046	0.0921	0.0052	Lipids
Xylonate	0.72	0.57	0.0029	7.82E-06	0.0519	0.0002	Carbohydrate
Sphinganine	0.7	0.46	0.029.7	0.0003	0.0854	0.0012	Lipids
Arabonate	0.71	0.58	0.0011	2.91E-06	0.0519	0.0001	Carbohydrate
2-Hydroxyadipate	0.68	0.46	0.009	0.0004	0.0581	0.0012	Amino acid
Pantothenate	0.66	0.55	0.0016	4.65E-05	0.0519	0.0004	CPGECs
Guanosine-2′,3′-cyclic monophosphate	0.55	0.35	0.0075	0.0002	0.0581	0.0011	Nucleotide
Xylose	0.75	0.68	0.0034	1.38E-05	0.0519	0.0002	Carbohydrate
Ascorbate (vitamin C)	0.52	0.49	0.0051	0.0009	0.0519	0.0017	CPGECs
4-Hydroxycinnamate	0.71	0.49	0.0417	4.24E-05	0.0958	0.0004	Secondary metabolism
*N*-Monomethylarginine	0.93	0.66	0.7329	0.045	0.4931	0.0242	Amino acid
*N-*Acetylglucosamine	!200.75	0.66	0.0059	0.0005	0.0519	0.0012	Carbohydrate
Arginine	1.89	2.11	0.0116	0.0008	0.0581	0.0017	Amino acid
Leucine	0.69	0.56	0.0233	0.0012	0.0744	0.0022	Amino acid
1-Pentadecanoylglycerophosphocholine (15:0)	0.64	0.5	0.3964	0.1351	0.3403	0.0536	Lipids
Aspartylleucine	!200.75	0.57	0.0647	0.0058	0.1164	0.0059	Peptide
Glutamate	1.84	2.33	0.0149	0.0037	0.0672	0.0047	Amino acid
2-Hydroxyglutarate	0.69	0.6	0.0044	0.0003	0.0519	0.0012	Lipids
Glycerate	0.55	0.44	0.0048	5.70E-05	0.0519	0.0004	Carbohydrate
1-Stearoylglycerol (1-monostearin)	0.61	0.6	0.0316	0.0178	0.0862	0.0124	Lipids
Cryptochlorogenic acid	1.07	!501.46	0.3528	0.0907	0.3193	0.0385	Secondary metabolism
Rhamnose	0.68	0.65	0.0009	7.87E-05	0.0519	0.0005	Carbohydrate
Nicotinate	0.67	0.62	0.0031	0.0007	0.0519	0.0017	CPGECs
Guanosine	0.94	0.79	0.3251	0.0009	0.3037	0.0017	Nucleotide
Ribose	0.72	0.67	0.0054	0.0017	0.0519	0.0028	Carbohydrate
Adenosine-2′,3′-cyclic monophosphate	0.61	0.41	0.0178	0.0004	0.0706	0.0012	Nucleotide
Pyruvate	0.66	0.61	0.022	0.0099	0.0732	0.0082	Carbohydrate
Tyrosine	!200.78	0.63	0.053	0.0029	0.1034	0.0038	Amino acid
Dihydroquercetin	0.29	0.51	0.0101	0.1426	0.0581	0.054	Secondary metabolism
Adenosine 2′-monophosphate (2′-AMP)	0.65s	0.53	0.0164	0.0022	0.0697	0.0033	Nucleotide

*Green: indicates significant difference (*p* ≤ 0.05) between the groups shown, metabolite fold of change < 1.00; Light Green: narrowly missed statistical cutoff for significance 0.05 < *p* < 0.10, metabolite fold of change < 1.00; Red: indicates significant difference (*p* ≤ 0.05) between the groups shown, metabolite fold of change ≥ 1.00; Light Red: narrowly missed statistical cutoff for significance 0.05 < *p* < 0.10, metabolite fold of change ≥ 1.00; Non-colored: mean values are not significantly different for that comparison; *p*-value and *q*-value indicate the significance and FDR of difference of the relative metabolite levels between D5-20 and D5-21 compared to D5-0, respectively. CPGECs, Cofactors, Prosthetic Groups, Electron Carriers.*

**FIGURE 4:**
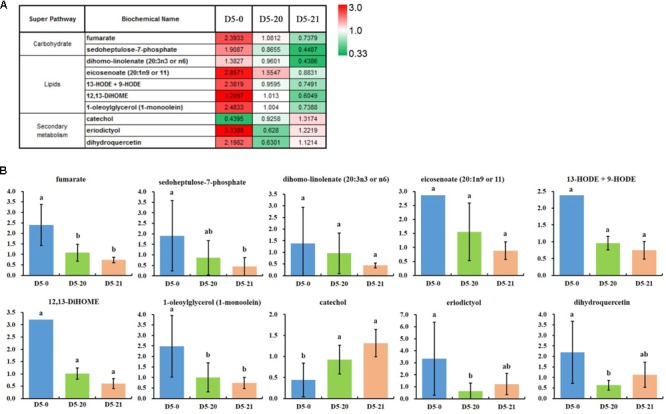
The 10 most variable compounds. **(A)** The ratio between maximum and minimum mean values for the most variable compounds by variety (>3-fold mean range). **(B)** The relative abundance of the 10 variable compounds. The ordinates represent scaled intensity of the relative abundance. Error bars are shown for six replicates of the relative abundance. Different letters indicate a statistically significant difference when analyzed by one-way ANOVA and a multiple comparison using Duncan’s test at *p* ≤ 0.05. For column diagram with no standards deviations, eicosenoate (20:1n9 or 11), 13-HODE + 9-HODE and 12,13-DiHOME in D5-0 line, the standards deviations are not shown due to the large variation in the presence of one to two biological replicates and the standards deviations are greater than the average value.

We compared the spread of the mean values across the lines, calculated the ratio between maximum and minimum mean values for all compounds, and selected compounds with a variation higher that threefold variation in the ratio between maximum and minimum mean values (**Figure 4A**). Oxidized lipids were highly variable and higher in D5-0. These may serve as messengers for communication both within and between cells, or may induce structural and metabolic changes in the cell of cambium.

In accordance with the RF data, cambium data were dominated by a disproportionate number of oxidized lipids (e.g., dihomo-linoleate, eicosenoate, 13-HODE+9-HODE, 12,13-DiHOME, and 1-oleoylglycerol) which were highest in D5-0. It is interesting that the secondary metabolite catechol was low in D5-0 line compared to two transgenic lines, while the two flavonoids eriodictyol and dihydroquercetin were high in this same line (**Figure 4B**).

### Compounds Associated With Cell Wall Metabolism

**FIGURE 5:**
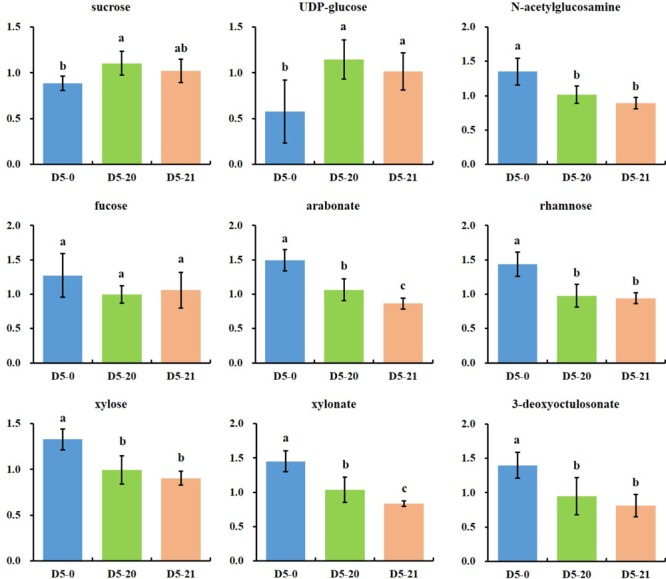
Metabolites associated with cell wall and polysaccharide metabolisms. The ordinates represent scaled intensity of the relative abundance. Error bars are shown for six replicates of the relative abundance. Different letters indicate a statistically significant difference when analyzed by one-way ANOVA and a multiple comparison using Duncan’s test at *p* ≤ 0.05.

We found pentose derivatives in our cambium RF analysis. **Figure 5** shows a wide range of compounds associated with cell wall metabolism. Several pentoses or pentose acids (fucose, arabonate, rhamnose, xylose, and xylonate) were increased in variety D5-0. At the same time, sucrose and UDP-glucose, the major precursors for carbon in the cell wall pathways, were lower in D5-0 compared to the two transgenic lines D5-20 and D5-21.

## Discussion

The cambium is a tissue unique to woody plants that consists of several layers of narrow elongated, parenchyma cells that are easily damaged during sampling. This high fragility makes traditional methods difficult to use for the study of cambial structure and function. The formation of cambium is directly related to the lateral growth of woody plants, so the metabolites in the cambium are crucial to improve wood growth and stress resistance in poplar. Therefore, the analysis of perennial poplar using UHLC/MS/MS or GC/MS may aid research into the composition and content of cambium metabolism. To understand the composition and variation of metabolites in non-transgenic and transgenic poplar, we performed standardized non-targeted UPLC–MS/MS and GC–MS metabolomic analysis.

Multi-gene transgenic poplar lines D5-20 and D5-21 exhibit greater wood growth, higher tolerance to multiple stressors (drought, salt, and waterlogging), and better insect tolerance than non-transgenic D5-0 in long-term greenhouse and field experiments (Su et al., 2011; Zhang et al., 2011). Transcriptomic analysis revealed that the expression of stress response genes changed in transgenic D5-20 line poplar (Zhang et al., 2014). All of the findings confirmed the effects of transgenes in transgenic poplars due to the introduction of multiple stress-related genes. In comparison these studies, we found small changes in metabolites associated with the transgenic lines to some extent.

Previous metabolomic studies focused on stress-related metabolic changes or the metabolic variation of species diversity during evolution and among different cultivars (Joshi et al., 2010; Schmidt et al., 2011; Hu et al., 2014; Jorge et al., 2016). Here, we focused on discovering the metabolites associated with multi stress-related genes transgenic lines and investigating the variations among different lines. Ultimately, we identified 197 metabolites with as significance difference between the three lines, in which the three major primary metabolism groups (amino acids, lipids, and carbohydrates) accounted for more than 70% of all identified metabolites. Levandi et al. (2008) used CE-MS [capillary electrophoresis (CE)] to compare the differences of metabolites between three *Bt* (*Bacillus thuringiensis*) transgenic lines and common maize, finding some metabolites with significantly differences in their content. Lin et al. (2014) identified 104 metabolites in significantly different amounts among different soybean cultivars, with a large number of these metabolites being amino acid, carbohydrate, or lipid metabolisms in essential metabolic processes in soybean seed development. Meanwhile, metabolome analysis indicated the content of these three main metabolites (amino acids, organic acids, and sugars) changed in response to water deficiency in two spring-wheat cultivars (Michaletti et al., 2018).

Previous report indicated that rapid and profound changes in amino acid pools in response to oxidative stress in rice and wheat (Shingaki-Wells et al., 2011). Here, we found two significantly increased metabolites (arginine and glutamate) in amino acid primary pathway in multi-genes transgenic lines compare to non-transgenic line. Arginine is the precursor of PA and NO (as important messenger molecules in organism) biosynthesis, which participate in almost all physiological and biochemical processes, including growth and development, stress resistance and other processes in plants (Crawford, 2006; Morris, 2006). However, arginine is negatively correlated with heading time, one of shared metabolite-morphological trait associations between *japonica* and *indica* rice (Hu et al., 2014). This is in disagreement with our results, where arginine was significantly increased in transgenic lines. Actually, arginine level is strongly associated with field phenotypes (height, diameter, and composite leaf angle), the *NAC154* overexpressing tissues verified the role of increased arginine levels in anti-senescence/dormancy-associated processes in poplar (Jervis et al., 2015). Glutamate is an important branching points for the biosynthesis of several amino acids, and serves as amino group donors for amino acid metabolism (Ishikawa et al., 2010). Moreover, glutamate induces the expression of *bHLH* and *IRO2*, both involved in regulation of stress responses, in rice roots (Kan et al., 2015). Therefore, increased arginine and glutamate levels might be responsible for better growth performances and stress-resistance in transgenic lines.

Among the carbohydrates metabolites which showed different levels in the transgenic lines, sucrose and UDP-glucose, both of which are major precursors associated with cell wall pathways, and both showed an increase in the two transgenic lines. A previous study reported that more than 62% carbohydrates, including sucrose, have shown significantly variations among 29 different soybean cultivars (Lin et al., 2014). Conversely, in maize kernels, there are no significantly differences among 14 maize varieties for over 80% of carbohydrates analyzed (sucrose included) (Rao et al., 2014). As the main photosynthetic product and the initial form of sugar transport, sucrose is hydrolyzed into glucose and fructose or sucrose synthases, and finally to form UDP-glucose and fructose in higher plants (Koch, 2004). However, seasonal cessation of wood formation is relevant to the reduced levels of sucrose in poplar (Deslauriers et al., 2009). GC–MS analysis shows that salt-tolerant varieties maize hybrids with different salt tolerance accumulate glucose, fructose, and sucrose (Richter et al., 2015). Additionally, the high levels sucrose detected in water-stressed leaf tissue of *Populus tomentosa* (Nishizawa et al., 2008), and the accumulation of sucrose, glucose, and fructose in *Populus* hybrids in response to drought (Kozlowski and Pallardy, 2002) suggest that some of these compounds are likely to act as osmotic agent to maintain cell turgor and stabilize cellular proteins (Seki et al., 2007). On behalf of a vital branch point in carbohydrate metabolism, UDP-glucose can synthetize other nucleotide sugars like UDP-glucuronic acid and directly guides synthesis of sucrose, starch, hemicellulose or cellulose, and pectin (Gibeaut, 2000; Bar-Peled and O’Neill, 2011). We observed an increased the levels of UDP-glucose associated with cell wall and polysaccharide metabolism in D5-20 and D5-21 lines, which is related to the superior performance of these transgenic lines. Previous study show that expression of *SacB* in transgenic sugar beet display increased levels of fructan (Bartels and Sunkar, 2005), while transgenic potato has higher content of the non-structural carbohydrates fructan, fructose, glucose, starch, and sucrose (van der Meer et al., 1994). These results were in agreement with our study, showing that *SacB* expression affects carbohydrates metabolism.

In contrast to the evident changes in amino acid and carbohydrate metabolites, we detected just a small percentage of secondary metabolites (about 10%), including alkaloids, benzenoids, flavonoids, and phenylpropanoids. Among of these, the most significant difference was in the content of catechol between transgenic lines and non-transgenic line. Catechol is readily oxidized by polyphenol oxidase (PPO), a process which increases the content of the anti-insect activity phenolics compounds producing highly reactive *o*-quinones (Duffey and Felton, 1991), and catechol itself also has anti-insect activity (Duffey and Stout, 1996). Haruta et al. (2001) propose that PPO contributes to aspen defense by enhancing the toxicity of catechol, and that release of catechol and PPO oxidation is a possible mechanism of toxicity in trembling aspen (*Populus tremuloides*). Indeed, approximately 50% of synthetic catechol is used to consume for pesticides (Qu et al., 2015). Therefore, catechol in transgenic lines has likely a role in insect resistance. *BtCry3A*, encoding a δ-endotoxin from *Bacillus thuringiensis*, and *OC-I*, encoding the proteinase inhibitor oryzacystatin I from riceconferring tolerance to coleopterous insecta, are present in both D5-20 and D5-21. So far, no reports are available on an interaction between *BtCry3A* or *OC-I* and catechol, but the overexpression of two genes and the increased catechol content are likely responsible to confer resistance to coleopterous insect.

Phenolic glycosides are one of the main secondary metabolites in poplar, and have been found in various poplar species. In *P. tremuloides*, four kinds of structure-related phenolic glycosides, salicin, salicortin, tremuloidin, and tremulacin are present, of these, salicin and salicortin are common in poplar, while grandidentoside, and HCH-salicorin have a relatively narrow distribution (Tsai et al., 2006). Metabolic profiling showed that *PdUGPase2* reduces the levels of sugar and starch, but increased phenolic compounds in *PdUGPase2* overexpressing *Populus* (Payyavula et al., 2014). Here, we detected the presence of salicin, but its differences between the three lines were not significant, and therefore we did not consider this compound for further experiments.

In summary, despite biological replicate variation, we were able to document significant metabolomic differences in the cambium of transgenic and non-transgenic poplar. The greatest differences between lines were two alpha-ketoglutarate derivatives and catechol that relevant to growth and insect-resistance, as well as sugars and its derivatives mostly associated with cell wall metabolism. As the cambium is the site of xylem production, these results are not surprising. However, evident difference in the content of these compounds between these poplar lines suggests that these metabolites may be important for stress response.

## Conclusion

In this study, we investigated the metabolome of the cambium of one non-transgenic and two multi-gene transgenic lines of hybrid poplar using both GC–MS and UPLC–MS/MS with a large-scale non-targeted metabolomics analysis. One hundred ninety-seven metabolites showed significant differences between the non-transgenic and multi-gene transgenic lines. Two alpha-ketoglutarate derivatives of arginine and glutamate in the amino acid primary pathway showed higher level in both transgenic lines, promoting growth and resisting to external stress. Moreover, we also found differences in the content of sucrose and UDP-glucose, mostly associated with major carbon precursors for cell wall pathways, and catechol, which relates to insect resistance. Our findings may provide a basis for further studies on cambium metabolism in poplar, molecular breeding to enhance stress-related activity.

## Author Contributions

KN and CD projected and implemented all the experiments and drafted the manuscript. XS, WZhu, WZhang, YD, and YS were involved in devising and directing the experiments and proofreading the manuscript. XS contributed to the concept of the research, gave constructive advice on the experiments, and finally completed the manuscript.

## Conflict of Interest Statement

The authors declare that the research was conducted in the absence of any commercial or financial relationships that could be construed as a potential conflict of interest.

## Footnotes

1 http://www.plantcyc.org

2 http://www.genome.jp/kegg/compound/
